# isma: an R package for the integrative analysis of mutations detected by multiple pipelines

**DOI:** 10.1186/s12859-019-2701-0

**Published:** 2019-02-28

**Authors:** Noemi Di Nanni, Marco Moscatelli, Matteo Gnocchi, Luciano Milanesi, Ettore Mosca

**Affiliations:** 1 Institute of Biomedical Technologies, Italian National Research Council, Via Fratelli Cervi 93, 20090 Segrate, MI Italy; 2 Department of Industrial and Information Engineering, University of Pavia, Via Ferrata 5, 27100 Pavia, Italy

**Keywords:** Somatic mutations, Next-generation sequencing, Cancer, Data integration

## Abstract

**Background:**

Recent comparative studies have brought to our attention how somatic mutation detection from next-generation sequencing data is still an open issue in bioinformatics, because different pipelines result in a low consensus. In this context, it is suggested to integrate results from multiple calling tools, but this operation is not trivial and the burden of merging, comparing, filtering and explaining the results demands appropriate software.

**Results:**

We developed *isma* (*i*ntegrative *s*omatic *m*utation *a*nalysis), an R package for the integrative analysis of somatic mutations detected by multiple pipelines for matched tumor-normal samples. The package provides a series of functions to quantify the consensus, estimate the variability, underline outliers, integrate evidences from publicly available mutation catalogues and filter sites. We illustrate the capabilities of isma analysing breast cancer somatic mutations generated by The Cancer Genome Atlas (TCGA) using four pipelines.

**Conclusions:**

Comparing different “points of view” on the same data, *isma* generates a unique mutation catalogue and a series of reports that underline common patterns, variability, as well as sites already catalogued by other studies (e.g. TCGA), so as to design and apply filtering strategies to screen more reliable sites. The package is available for non-commercial users at the URL https://www.itb.cnr.it/isma.

**Electronic supplementary material:**

The online version of this article (10.1186/s12859-019-2701-0) contains supplementary material, which is available to authorized users.

## Background

The identification of somatic mutations from Next Generation sequencing (NGS) data is a challenging task. Several studies compared the single nucleotide variations (SNVs) [1–3] and insertions/deletions (INDELs) [4, 5] detected by different computational tools and underlined relevant discrepancies. Therefore, it is recommended to analyse the same NGS data using multiple callers, like Mutect [6], SomaticSniper [7] and Varscan [8], which generate lists of mutations encoded in Variant Call Format (VCF) [9]. This way of facing conflicting predictions demands appropriate tools that harmonize different outputs and enable comparative analyses [4]. Indeed, for instance, mutation callers encode the same information in multiple ways (Table 1) and generate outputs with relevant qualitative (e.g. germline/somatic/loss-of-heterozygousity, SNVs/INDELs) and quantitative (number of sites found) differences. More generally if, in principle, the use of multiple callers is expected to reduce false positive findings, in practice, the resulting large and heterogeneous lists of mutation sites increase the complexity of the subsequent interpretations. Existing tools like myVCF [10], NGS-pipe [11], VariantTools [12], vcfR [13] and VCFTools [9], implement functions and pipelines to work with VCF files, but do not specifically address the problem of integrating and comparing the results of different mutation callers. A few tools exist to address this problem: Cake [14] (a bioinformatics pipeline implemented in perl) offers the opportunity to run multiple callers and applies customizable filtering steps to obtain a final unique list of single nucleotide variations (SNVs); BAYSIC [15] (implemented in perl) provides a bayesian method for combining SNVs from different variant calling programs.

**Table 1:** Pipelines for somatic mutation call from matched tumor-normal samples

		Mutect [6]	Mutect (v2) [6]	Muse [22]	SomaticSniper [7]	Strelka [23]	Varscan (v2) [8]
Variant type		SNV	SNV, INDELs	SNV	SNV	SNV, INDEL	SNV, INDEL
Mutation inheritance		Somatic	Somatic	Somatic	Germline, somatic, LOH	Somatic	Germline, somatic, LOH
Model		Bayesian	Bayesian	Bayesian Markov	Bayesian	Bayesian	Fisher’s exact statistics
Implementation		Java	Java	C/C++	C	Perl	Java
Allelic Depth^a^	field(s)	AD	AD	AD	BCOUNT	AU:CU:GU:TU	AD and RD
	value(s)	2 comma separated numbers	2 comma separated numbers	2 comma separated numbers	4 comma separated numbers	4 comma separated numbers	2 numbers
License		Freely available for academic, non-commercial research purposes	Beta status; not available for commercial/for-profit licensing	GNU GPL V2	MIT	GNU General Public License	Free for non-commercial use by academic, government, and non-profit/not-for-profit institutions

(^a^) The way in which the allelic depth (number of reads supporting an allele) is encoded in VCF files is reported as an example of heterogeneity among pipeline outputs

**Fig. 1:**
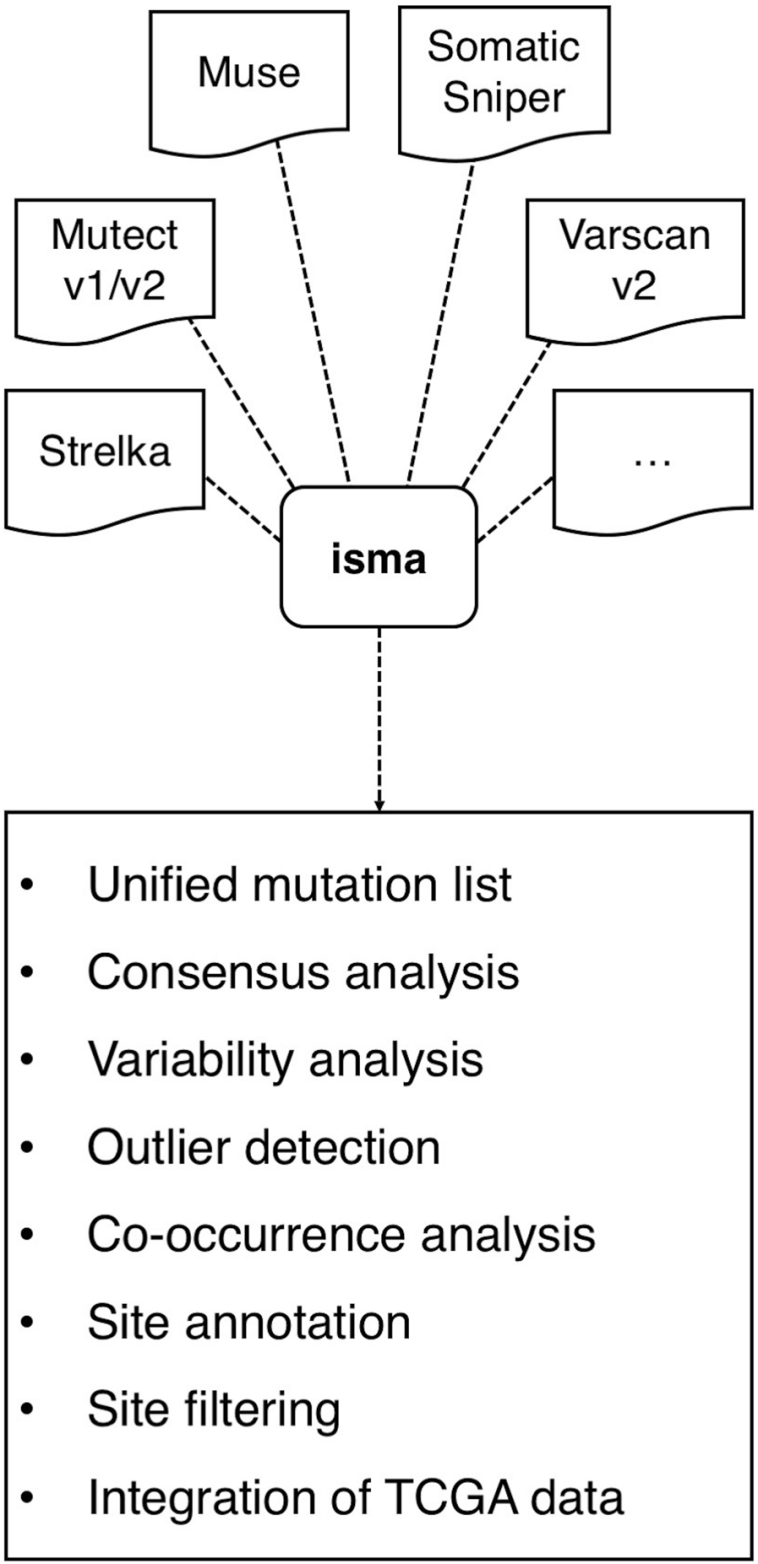
Overview of isma. Integrative analysis of somatic mutations detected by multiple pipelines

Here, we describe *isma* (*i*ntegrative *s*omatic *m*utation *a*nalysis), an R package that provides functions for the joint analysis of VCF files generated by somatic mutation callers from NGS data (Fig. 1). Differently from existing tools, beyond site integration and filtering, *isma* provides functions for a more in-depth analysis of mutation sites occurrence across subjects and tools, considering both SNVs and INDELs. The results generated by isma underline common patterns (e.g. recurrent calls, tool consensus in each subject), specificities (e.g. outlier samples, pipeline specific sites, genes enriched in calls from a single pipeline), as well as sites already catalogued by other studies (e.g. The Cancer Genome Atlas (TCGA) [16]), so as to design and apply filtering strategies to screen more reliable sites.

## Implementation

The software isma is implemented in R. The package takes in input mutation sites encoded in VCF files or tab-delimited text files. isma extracts mutation site information from the output of multiple mutation callers by means of specific parsers and integrates sites into a unique data structure:

- mut_sites <− pre_process (“config.txt”)

Most of the analyses can be easily carried out through a few wrapper functions, like site_analysis and gene_analysis for site- and gene-level analyses respectively. Nevertheless, many routines are available as part of the user interface to carry out custom analyses (Table 2). Gene-level analyses require mutation site annotation, for which isma relies on the R package VariantAnnotation [17] or, alternatively, on user-provided files. Computationally demanding analyses (e.g. the comparison among all-pairs of hundreds of subjects) are implemented in parallel, using the support provided by the R package parallel. The package isma contains a tutorial available as R vignettes:

- vignette(“isma”)

**Table 2:** isma user interface

Function name	Description
pre_process	Read and integrate input files; generate unique identifiers
site_analysis	Perform site-level analyses, calling get_sites_statistics, overlap_Tools, overlap_Subjects
gene_analysis	Perform gene-level analyses, calling get_sites_statistics, overlap_Tools, overlap_Subjects, gene_mutation
site_annotation	Perform site annotation
integrate_TCGA	Integrate mutation evidence from TCGA
consensus_Tools	Calculate the consensus among tools
get_sites_statistics*	Calculate the co-occurrence of mutation sites/genes across callers and subjects
overlap_Subjects*	Calculate subject-by-subject site/gene co-occurrence matrix
overlap_Tools*	Calculate tool-by-tool site/gene co-occurrence matrix
ese_allsubj*	Calculate the variation of site/genes amount and show the results for each tool
ese_tool_subj*	Calculates the variation of site/genes amount, considering separately each tool and returns the results for each subject
ese_subj_tool*	Calculates the variation of site amount, considering separately each subject and returns the results for each caller
calculate_dist_to_exon	Calculate the site distance from the nearest exons
gene_mutation	Calculate the gene-by-subject mutation matrix and the gene mutation frequency vectors
filtering_sites	Filter sites

The asterisk (*) indicates functions that work both at site- and gene-level

## Results

In this section, we will describe isma considering breast cancer (BC) mutations from TCGA, collected using the function get_TCGA_sites. In particular, we considered mutation profiles of 975 subjects detected by four variant callers: Mutect2, Varscan2, Muse and SomaticSniper (Additional file 1).

- mut_sites <- get_TCGA_sites (tools = c("muse", "mutect2", "varscan2", "somaticsniper"), n_subjects = 975)

Note that these sites were already filtered by TCGA and are therefore less noisy than the corresponding initial variant caller outputs that would constitute the input of isma in a typical use scenario. Nevertheless, the exploratory analyses made possible by isma underlined interesting patterns even among such filtered calls from TCGA.

The analyses presented below can be easily run by means of site_analysis and gene_analysis wrapper functions and include quantification of site/gene occurrence across callers and subject, consensus among tools, detection of outlier subjects and tools, variation of detected sites at different cut-offs on alignment results (e.g. read depth) and integration of information from TCGA.

### Site occurrence across callers and subjects

The co-occurrence of sites across tools and subjects is quantified by get_sites_statistics. This operation allows the user to quantify the fraction of tool-specific calls, the distribution of the sites across tools in each subject and tool consensus on sites. These results are used to detect and mark outlier features (subjects and tools), defined by the inter-quartile range (Tukey’s fences) (Table 3). The amount of shared sites between each pair of callers and subjects is calculated and organized, respectively, in callers-by-callers and subjects-by-subjects site co-occurrence matrices by the functions overlap_Tools and overlap_Subjects. Site co-occurrence matrices are used to summarize consensus and dispersion. Caller consensus relative to a subject is quantified by means of the consensus score (CS), defined as the sum of ratios between the amount of co-occurring sites (off-diagonal elements of the tools-by-tools site co-occurrence matrix) and tool-specific calls (diagonal elements) normalized by the total number of possible tool pairs:

$$ \mathrm{CS}=\frac{\sum_i^n\left(\frac{1}{x_{i,i}}{\sum}_{j\ne i}^n{x}_{i,j}\right)}{P\left(n,2\right)} $$

where *n* is the number of tools, *x*_*i,j*_ are the sites shared between tools *i* and *j*, and *P*(*n*, 2) is the number of permutations of tools in pairs.

**Table 3:** Outlier subjects report

Subject	Hypermutated	Imbalance in the number of sites across tools	Imbalance in consensus among tools	Tool consensus score (CS)
A0JC	NO	YES	YES	YES
A1G6	NO	YES	YES	YES
A1LI	NO	YES	NO	YES
A0UO	YES	YES	YES	YES

Examples of subjects recognized as outliers according to the number of sites, imbalance in the number of sites across tools, imbalance in consensus among tools and tool consensus score

**Fig. 2:**
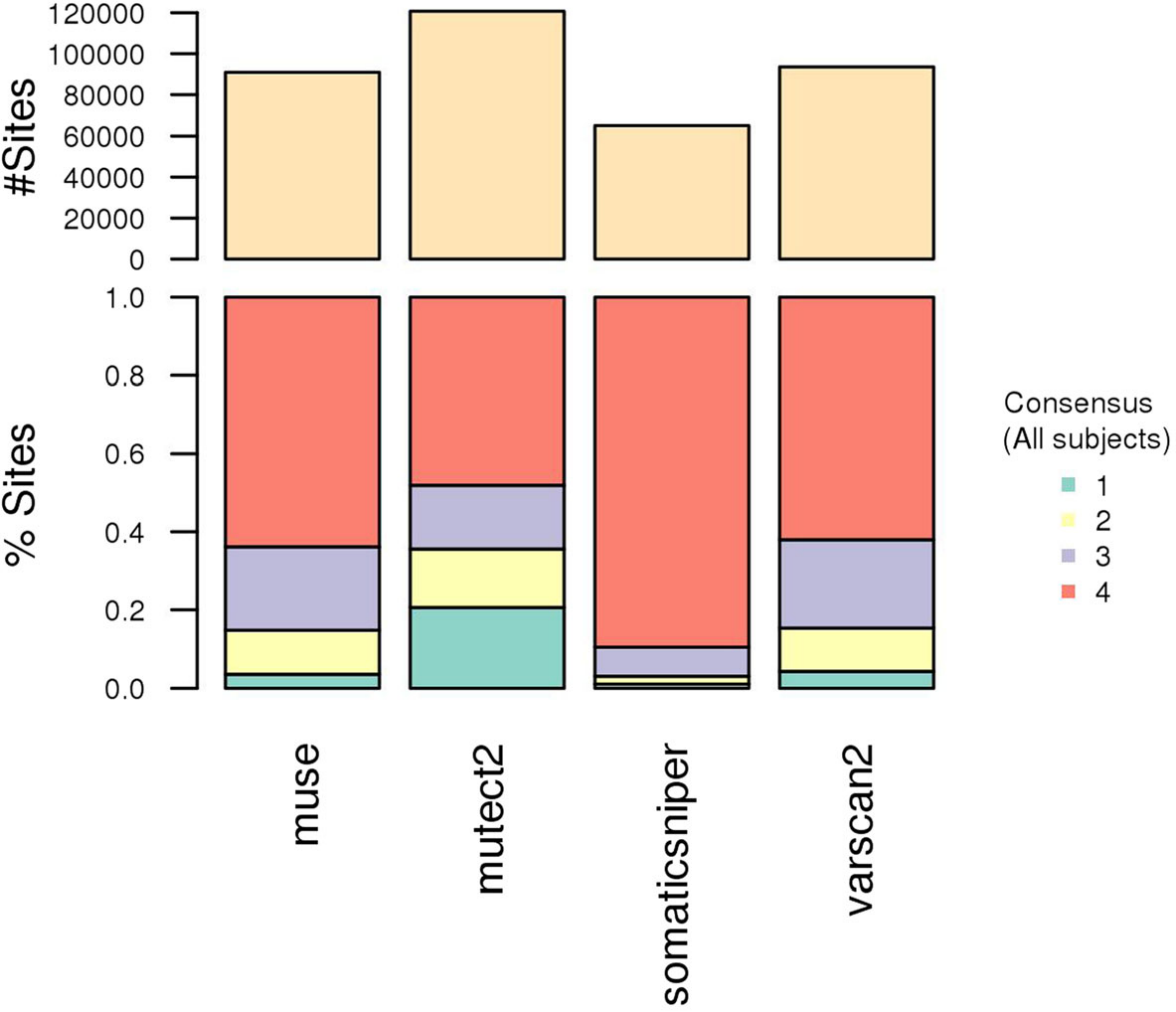
Global consensus plot. Overall consensus among pipelines; results obtained on BC mutations detected by TCGA in 975 subjects

**Fig. 3:**
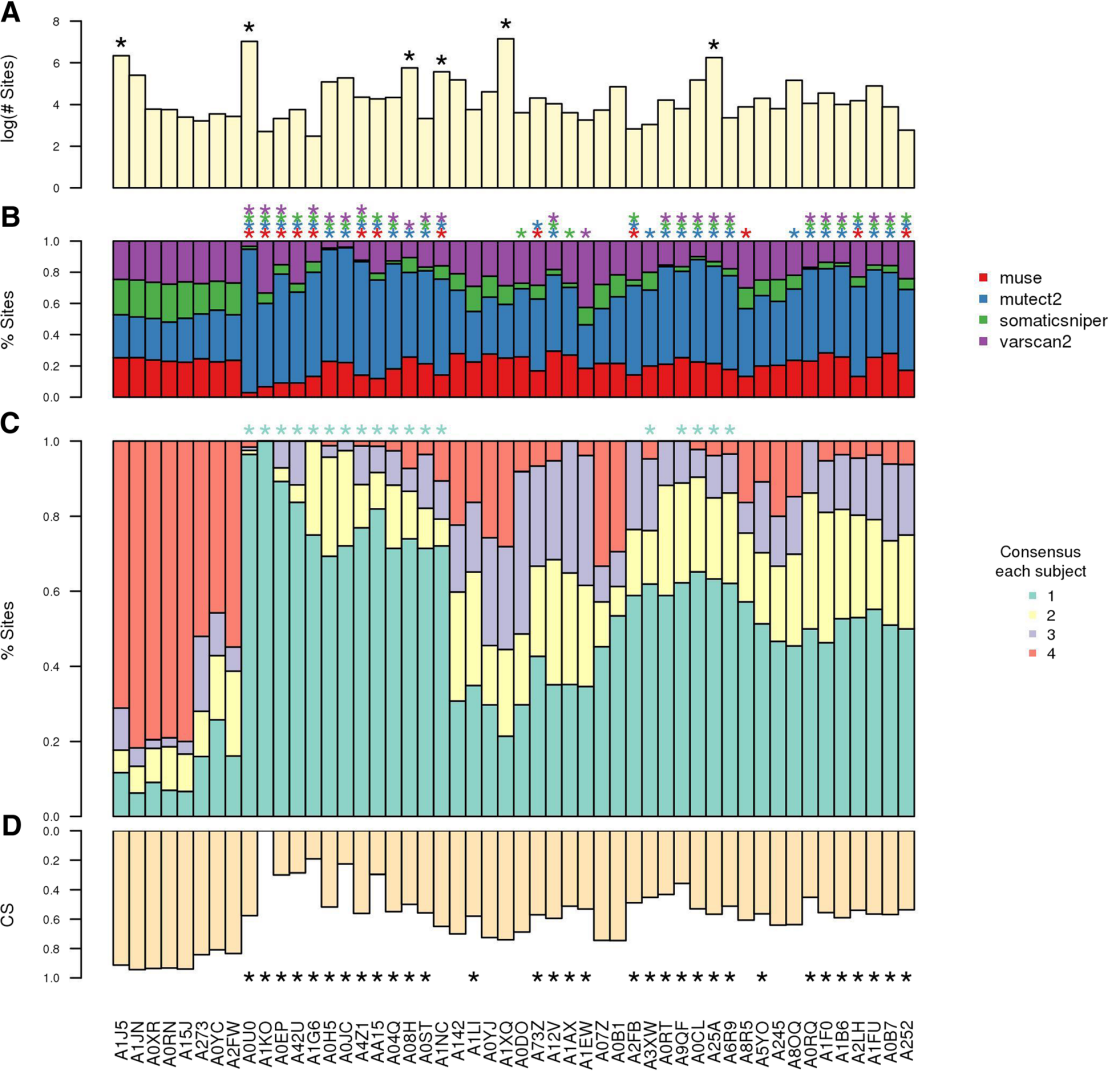
Detailed consensus plot. **a** Number of mutation sites. **b** Fraction of sites called by different pipelines. **c** Tool Consensus across subjects. **d** Consensus score (CS). **a-d** Asterisks indicate outliers. Results shown only for 50 subjects (out of 975), selected to include different types of outliers as well as samples without abnormal behaviours (Additional file 1)

The results of these analyses are summarized into consensus plots, co-occurrence matrices plot and a series of text files, like the summary table of outlier subjects. The overall consensus plot (Fig. 2) reports the total number of sites found by each tool and the fraction of calls shared among tools. Note how mutect2 found the highest number of sites, the 50% of which was not reported by other callers (Fig. 2). The consensus plot per subject shows the total number of unique sites, the fraction of sites found by each tool, the distribution of the consensus across subjects and the CS (Fig. 3). Note the presence of a few hypermutated subjects (i.e. A1XQ, A0U0, A08H, A1J5, A1NC and A25A) (Fig. 3a). Several subjects display an imbalance of calls among the pipelines (Fig. 3b). Further, there are subjects with a relevant (e.g. A1J5 and A0XR) or poor (e.g. AIKO and A0JC) proportion of sites supported by more than one caller (Fig. 3c). Lastly, note how CS underlines, by means of a unique score, subjects with issues in tool consensus, including imbalances in the number of sites or consensus among tools (Fig. 3d and Table 3).

**Fig. 4:**
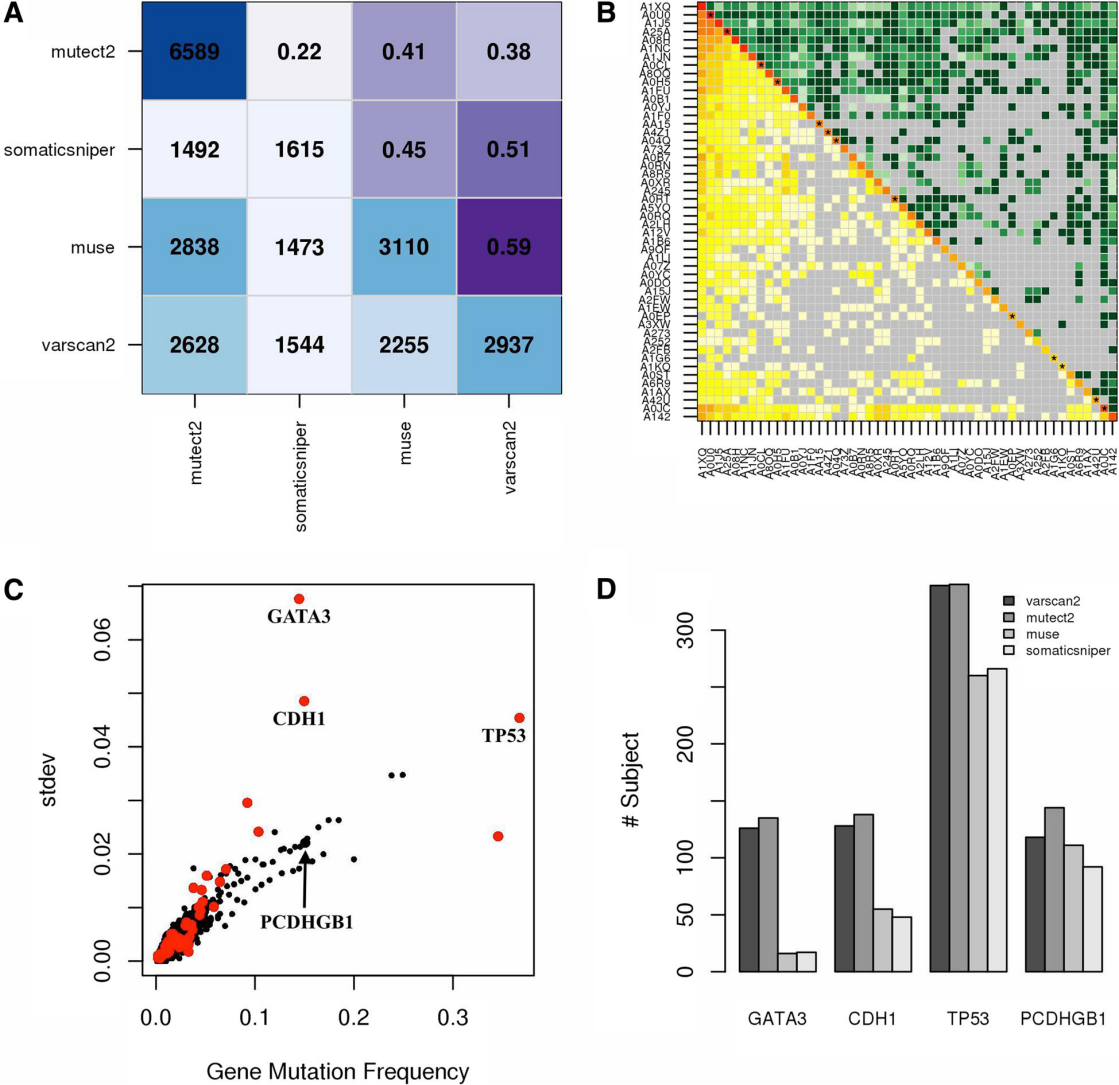
Site co-occurrence plots and gene mutation frequency variability. **a** Total number of sites (diagonal), site co-occurrence among mutation callers (below diagonal) and corresponding similarity between callers (Jaccard index, above diagonal). **b** Total number of mutated genes (diagonal), mutation co-occurrence across subjects (below diagonal) and corresponding coefficients of variations (CVs) across pipelines (above diagonal). Asterisks indicate CVs greater than 1; grey colour indicates no mutation co-occurrence between two subjects. **c** Standard deviation of gene mutation frequency across pipelines; red: genes associated with BC [19–21]. **d** Number of subjects with mutations detected by each tool. **a-b** Results obtained on BC mutations from 50 subjects (Additional file 1); **c-d** results obtained on all 975 subjects (Additional file 1)

Site co-occurrence between callers revealed that mutect2 detected up to 3 times more sites than other tools, while muse and varscan shared approximately the 60% of their sites (Fig. 4a). The mutation co-occurrence in each pair of subjects underlines similarities between mutation profiles; this information is completed with an estimation of the variability (coefficient of variation) of such co-occurrences due to the use of different callers (Fig. 4b). The package provides the possibility of calculating, for every gene, the fraction of subjects with at least one mutation, i.e. the gene mutation frequency across subjects (*f*), and its dispersion across callers. The corresponding plot, obtained on BC TCGA sites, underlined the presence of some genes, including known BC genes as GATA3 and CDH1, with a particularly higher variation of *f* (Fig. 4c): indeed, mutect2 and varscan2 detected much more sites than other callers in GATA3 and CDH1 (Fig. 4d).

### Called sites and sequencing results

The variation of caller output at different cut-offs on site-level quantities (e.g. *minimum* number of reads, allele frequency) is informative of caller performance and samples (subjects) specificities. This analysis can be done by the function:

- ese1 <- ese_allsubj(mut_sites$sites, type = “Site”)

**Fig. 5:**
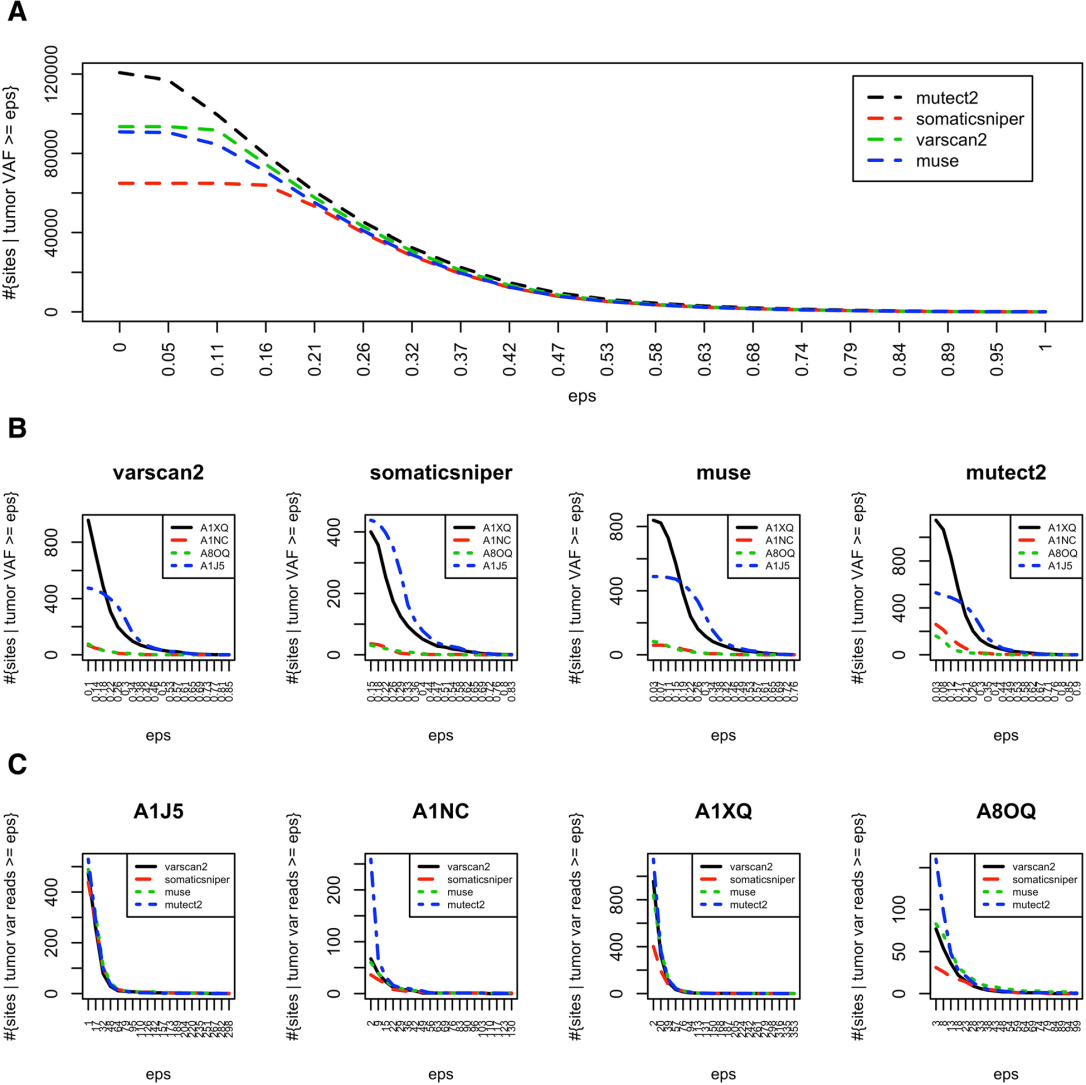
Number of called sites at various filtering criteria. Number of mutation sites at varying tumor VAF for (**a**) the whole dataset (975 subjects) and (**b**) in single subjects. **c** Number of sites at varying number of reads supporting the alternative allele in four subjects. **a-c** Results obtained on BC mutations detected by TCGA

The pipelines used to call mutations in TCGA BC data show a different behaviour, especially at low tumor variant allele frequency (VAF). In fact, in this range, mutect2 calls more sites than other tools, SomaticSniper detects almost half of mutect2 sites, while muse and varscan2 show similar trend and are halfway between mutect2 and SomaticSniper (Fig. 5a). This global pattern is particularly relevant in some subjects (Fig. 5b-c).

### Collecting data from the TCGA

The function integrate_TCGA uses the R package TCGAbiolinks [18] to collected data from the TCGA. These data are used to support the mutation sites under analysis with the possible evidence of availability of the same sites among those already catalogued at TCGA, which would be an additional evidence of site reliability.

## Conclusions

The R package isma provides functions for the integrative analysis of mutation sites detected by multiple pipelines. It quantifies the consensus between somatic mutation call pipelines, estimates pipeline variability and biological variability, and underlines outlier features (subject/tools) that may require further investigation. Indeed, an outlier subject may reflect a biological phenomenon (e.g. due to tumor genetic heterogeneity) and/or an experimental problem (e.g. poor biological sample, sequencing performance). The application of isma on BC mutations from TCGA underlined relevant variations among pipelines across subjects, with extreme cases characterized by a very poor consensus. Relevant imbalances among pipelines were also spotted at gene level, which implies a significant variability in the estimation of gene mutation frequency according to the pipeline used. In general, mutect2 reported a higher number of sites at low VAF in comparison to other callers.

In conclusion, the knowledge emerging from the analyses made possible by isma is useful to screen more reliable mutation sites, carry out comparative analysis among pipelines and, lastly, may suggest novel biological insights.

## Availability and requirements

Project name: isma

Project home page: https://www.itb.cnr.it/isma

Operating system: Platform independent

Programming language: R (> = 3.3.3)

Other requirements: The R Project for Statistical Computing.

License: GNU General Public License (> = 2)

Any restrictions to use by non-academics: According to GNU General Public License (> = 2)

## Additional file

Additional file 1: TCGA barcodes. List of TCGA barcodes used in this study. (TXT 33 kb)

## Abbreviations

BC: Breast cancer
INDEL: Insertions, deletions
isma: Integrative somatic mutation analysis
NGS: Next generation sequencing
SNV: Single nucleotide variations
TCGA: The cancer genome atlas
VCF: Variant Call Format
